# Role of Maleic Anhydride-Grafted Poly(lactic acid) in Improving Shape Memory Properties of Thermoresponsive Poly(ethylene glycol) and Poly(lactic acid) Blends

**DOI:** 10.3390/polym14183923

**Published:** 2022-09-19

**Authors:** Wasana Nonkrathok, Tatiya Trongsatitkul, Nitinat Suppakarn

**Affiliations:** 1 School of Polymer Engineering, Institute of Engineering, Suranaree University of Technology, Nakhon Ratchasima 30000, Thailand; 2 Research Center for Biocomposite Materials for Medical Industry and Agricultural and Food Industry, Suranaree University of Technology, Nakhon Ratchasima 30000, Thailand; 3 Center of Excellence in Biomechanics Medicine, Suranaree University of Technology, Nakhon Ratchasima 30000, Thailand; 4 Center of Excellence in Microbial Technology for Agricultural Industry, Suranaree University of Technology, Nakhon Ratchasima 30000, Thailand

**Keywords:** poly(lactic acid), poly(ethylene glycol), shape memory polymer, compatibilizer

## Abstract

Generally, poly(ethylene glycol) (PEG) is added to poly(lactic acid) (PLA) to reduce brittleness and improve mechanical properties. However, shape memory properties of PEG/PLA blends suffered due to the blend’s incompatibility. To enhance shape memory abilities of the blends, 0.45% maleic anhydride-grafted poly(lactic acid) (PLA-*g*-MA) was used as a compatibilizer. Thermal and mechanical properties, morphologies, microstructures, and shape memory properties of the blends containing different PLA-*g*-MA contents were investigated. The compatibilized blend with 2 wt% PLA-*g*-MA exhibited enhanced tensile modulus, strength, and elongation at break, as well as a lower glass transition temperature and degree of crystallinity than the uncompatibilized blend. Results revealed that PLA-*g*-MA improved interfacial adhesion between phases and promoted chain entanglement. Shape fixity performance of the compatibilized blends were comparable to that of neat PLA. The compatibilized blend containing 2 wt% PLA-*g*-MA possessed the best shape fixity and recovery performance. Although a high recovery temperature was expected to enhance the recovery of the PEG/PLA blends, the compatibilized blends can be recovered to their original shape at a lower temperature than the PLA. This study illustrated the possibility of optimizing PLA properties to meet requirements necessary for biomedical applications.

## 1. Introduction

Shape memory polymers (SMPs) have attracted considerable attention over the last decades due to their light weight, ease of fabrication, and ability to retain their initial shape in the presence of a stimulus. They can be formed into the temporary shape and then restored to their permanent shape in response to an external stimulus [1,2,3,4], such as heat [5,6], light [7], electrical field [8], magnetic field [9], pH [10], solvent [11], etc. Polymeric materials, in general, undergo a noticeable change in physiochemical properties as a function of temperature. As a result, thermally induced SMPs are the most common and extensively studied [5,6]. These SMPs can be triggered directly by heat, such as hot gas or warm water.

SMPs are typically comprised of two components: a hard segment and a soft segment. The hard segment acts as netpoints that memorize the permanent shape via physical or chemical cross-linking. The soft segment, also known as the reversible phase, acts as a switch, allowing the molecular chains to temporarily deform and is responsible for the shape recovery process [12]. A temporary shape of SMPs is achieved through applying mechanical force, while heating above its transition temperature (T_trans_), i.e., glass transition (T_g_) or melting temperature (T_m_) of the reversible part, which is referred to as programming temperature (T_p_). The deformed shape is maintained upon cooling down. The restoration of the permanent shape can be achieved by reheating above T_trans_ (called recovery temperature (T_r_)) [13,14,15,16].

Poly(lactic acid) (PLA) is a well-known biodegradable polyester with a wide range of applications, particularly in medical fields, due to its good mechanical strength, high biocompatibility, and nontoxicity [17,18]. Furthermore, several studies have revealed the use of PLA as a shape memory material with triggering temperatures close to its T_g_ of about 60 °C. At temperatures below its T_g_, PLA can be programmed into a temporary shape, and it returns almost entirely to its original shape at temperatures above its T_g_ [19,20,21]. However, PLA still has some drawbacks such as brittleness, and high T_g_, which may cause tissue damage in humans when used in some medical applications [22,23].

The addition of plasticizers is an interesting approach for tuning T_g_ and reducing the brittleness of PLA [24]. Poly(ethylene glycol) (PEG) is considered as an effective plasticizer for PLA due to its miscibility, biodegradability, and nontoxicity. Many research groups investigated the properties of PLA blended with PEG of molecular weights ranging from 1000 to 20,000 g/mol [25,26,27,28,29,30,31]. In general, the addition of PEG to PLA decreases T_g_ and increases elongation at break. Nonetheless, low molecular weight PEG has a tendency to migrate from the PLA matrix or evaporate during the process, and a PLA-PEG phase separation occurs when the PEG content exceeds a certain threshold. In addition, Guo et al. [28] reported that shape memory properties of PEG/PLA blends were marginally reduced when the PEG content was increased to 15 wt%, but significantly reduced when the PEG content was increased to 20 wt%. This was due to phase separation, which occurred in particular at a PEG content of 20 wt%.

Compatibility between two polymer components has a significant impact on the properties of a polymer blend and can be enhanced by introducing block or grafted copolymers with segments capable of physical or chemical interacting with the blend components [32,33]. Several studies have reported the use of maleic anhydride-grafted on polymer matrix as a compatibilizer to improve the compatibility of polymer blends [34,35,36,37]. Hwang et al. [35] studied effect of the maleic anhydride and DCP concentrations on the grafting and the properties of PLA. They found that T_g_ and percent crystallinity of the PLA decreased, but its mechanical properties remained unchanged. Hassouna et al. [36] investigated effect of low molecular PEG (MW = 400 g/mol) and maleic anhydride-grafted PLA (PLA-*g*-MA) copolymer on the ductility of PLA blends. Their findings revealed that the T_g_ of the PLA blend containing 20 wt% PEG decreased to 23 °C when compared to the T_g_ of the neat PLA at 60 °C. They also found that adding 10 wt% PLA-*g*-MA reduced the T_g_ of the PEG/PLA blend to 14 °C. In addition, Kim et al. [37] found that increasing the compatibility between two polymer phases enhanced the mechanical properties of the system as well as its shape memory behaviors. In their research, styrene–acrylonitrile-maleic anhydride (SAN-MAH) copolymer was used as a compatibilizer for poly(lactic acid) (PLA) and poly(methyl methacrylate-block-n-butyl acrylate-block-methyl methacrylate) (80/20) (PLA/Poly(MnBM)) blend. The shape recovery ratios for the PLA/Poly(MnBM) blends with and without the addition of 1 wt% SAN-MAH were 83 and 56%, respectively. Although the maleic anhydride-graft-poly(lactic acid) (PLA-*g*-MA) has been studied for its effect as a compatibilizer on the physical properties of PEG/PLA blends, the shape memory performance of the compatibilized materials has not yet been investigated.

In this study, we investigated the effect of the PLA-*g*-MA, used as a compatibilizer on shape memory abilities of PEG/PLA (10/90) blends. Our hypothesis was that by increasing the compatibility of PEG and PLA matrix, shape memory properties of the PEG/PLA blends such as shape recovery ratio (R_r_), shape fixity ratio (R_f_), and recovery rate would be improved. Additionally, thermal and mechanical properties, morphologies, and microstructures of the PEG/PLA blends containing different PLA-*g*-MA contents were respectively studied by differential scanning calorimetry (DSC), tensile test, scanning electron microscopy (SEM), and in situ synchrotron small-angle X-ray scattering and wide-angle X-ray scattering (SAXS and WAXS) microscopies.

## 2. Experimental

### 2.1. Materials

A semi-crystalline poly(lactic acid) (PLA) with a 4% D-isomer content, under the trade name Ingeo^TM^ biopolymer PLA, was purchased from Nature Works LLC (Minnetonka, MN, USA). Poly(ethylene glycol) (PEG) with a molecular weight of 4000 g/mol was supplied by Dow Chemical Company (Midland, MI, USA). Maleic anhydride (MA) was purchased from Loba Chemie Pvt. Ltd. (Mumbai, Maharashtra, India). Dicumyl peroxide (DCP) was purchased from Sigma-Aldrich Co., LLC (Burlington, MA, USA). Maleic anhydride-grafted poly(lactic acid) (PLA-*g*-MA) was prepared in house using PLA, MA and DCP.

### 2.2. Preparation of PLA-g-MA and PEG/PLA Blends

To prepare PLA-*g*-MA and PEG/PLA blends, a twin-screw extruder (Charoen Tut Model CTE-D16L32, Samutprakarn, Thailand) (L/D = 32) with a die diameter of 3 mm was used. The extruder temperature profile, from the feed throat to the die, was 50/160/180/180/180/180/175 °C. The screw speed used was 80 rpm (r/min). Rod extrudates were cooled by air and cut into small pellets using a pelletizer.

The PLA-*g*-MA compatibilizer was synthesized by grafting of poly(lactic acid) with maleic anhydride via reactive extrusion using the twin-screw extruder. PLA, DCP (1 phr), and MA (5 phr) were pre-mixed before being fed into the extruder. The obtained PLA-*g*-MA pellets were dried at 100 °C under a vacuum for 4 h to remove any unreacted MA.

To investigate influence of the compatibilizer on properties of the PEG/PLA blends, PLA and 10 wt% PEG, designated as PEG/PLA, were mixed with various PLA-*g*-MA contents, i.e., 2, 6 and 10 wt%. The samples were named according to the weight percentages of PLA-*g*-MA in the blends. For example, the PEG/PLA blend containing PLA-*g*-MA of 2 wt% was named 2PMA/PEG/PLA.

All test specimens were compressed molded at 175 °C for 10 min under a pressure of 100 MPa in a compression molding machine (LabTech Model LP20-B, Samutprakarn, Thailand) being allowed to cool at room temperature.

### 2.3. Characterizations of PLA-g-MA

The proportion of maleic anhydride grafted to PLA was determined using the titration technique. The dried PLA-*g*-MA sample was purified prior to use. At room temperature, the sample was dissolved in chloroform (RCI Labscan, Bangkok, Thailand) and magnetically stirred at 350 rpm to obtain a homogeneous solution. Methanol (RCI Labscan, Bangkok, Thailand) was then gradually added to the solution to re-precipitate it. Purified PLA-*g*-MA was filtered and dried in a vacuum at 80 °C for 4 h. To determine the amount of maleic anhydride grafted to PLA, the purified PLA-*g*-MA was dissolved in chloroform and titrated with methanolic potassium hydroxide (QREC, Chon Buri, Thailand) solution (KOH). The percentage of grafted maleic anhydride was calculated using Equation (1).

(1) $$\%\mathrm{MA}\;(\%)=\frac{\left(N_{\mathrm{KOH}}{\times V}_{\mathrm{KOH}}\times 98.06\right)}{{2W}_{\mathrm{sample}}}\times 100$$

where N_KOH_ is the normality of KOH (mol/L); V_KOH_, the volume of KOH (mL); W_sample_, the weight of sample (g); and the molecular weight of maleic anhydride is 98.06 g/mol.

Fourier transform infrared (FT-IR) (Bruker Tensor 27, Billerica, MA, USA) spectrometer with an attenuated total reflection (ATR) accessory was used to identify functional groups of PLA and PLA-*g*-MA. Spectrum of a sample was recorded in a wavenumber range of 4000 to 400 cm^−1^ with a resolution of 2 cm^−1^ and a number of scans of 64.

### 2.4. Characterizations of PEG/PLA Blends

#### 2.4.1. Tensile Testing

Tensile testing was carried out on a universal testing machine (UTM) (Instron 5565 Model, Norwood, MA, USA) with a load cell of 5 kN and a cross head speed of 10 mm/min. The test specimens were prepared in accordance with the ASTM D 638 standard (Type V). At least five specimens were tested, and the average value was reported.

The data from of the tensile test were statistically analyzed using IBM SPSS Statistic, version 24.0. (Armonk, NY, USA). The data was evaluated using one-way ANOVA and Turkey’s post hoc comparison test on three replication values (*n* = 3) from five examined specimens. To identify statistical differences between the comparison groups, the level of statistical significance was set at *p* < 0.5.

#### 2.4.2. Thermal Characteristics

Thermal characteristics i.e., glass transition temperature (T_g_), crystallization temperature (T_c_), cold crystallization temperature (T_cc_), melting temperature (T_m_), and crystallinity (X_c_) of specimens, before and after stretching, were determined using a differential scanning calorimeter (DSC) (Perkin Elmer, Waltham, MA , USA). To compare their actual thermal properties, unstretched and stretched samples were subjected to a single heating step between 25 and 200 °C at a rate of 10 °C/min under a nitrogen atmosphere. The crystallinity (X_c_) of each sample was estimated using the following Equation (2):

(2) $$X_{c}(\%)=(\frac{{\Delta H}_{m}{-\Delta H}_{\mathrm{cc}}}{{\omega \Delta H}_{m}^{o}})\times 100$$

where ${\Delta H}_{m}$ and ${\Delta H}_{\mathrm{cc}}$ are the enthalpies of melting and cold crystallization, respectively. ω is the weight fraction of PLA and ${\Delta H}_{m}^{o}$ is melting enthalpy of 100% crystalline PLA (93.7 J/g) [38].

#### 2.4.3. Morphology

Morphologies of the PEG/PLA blends were analyzed by using a scanning electron microscope (SEM) (JEOL JSM-6010LV, Peabody, MA, USA) at a voltage of 5 kV. Samples were cryo-fractured in liquid nitrogen and coated with gold for 3 min to ensure suitable electrical conductivity.

#### 2.4.4. Shape Memory Behaviors

Shape memory behaviors of the PEG/PLA blends were quantified using two parameters, R_f_ and R_r_. The shape fixity ratio (R_f_) quantifies the ability of a specimen to fix temporary deformations that occur during the programming process. The shape recovery ratio (R_r_) measures how well a specimen can return to its original shape.

**Figure 1:**
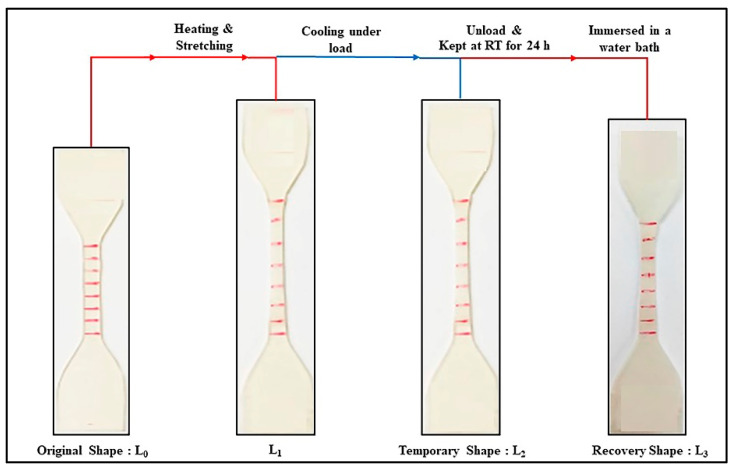
Diagram of a test specimen at different stages of the shape memory test.

Shape memory tests were performed using a universal testing machine (UTM) (Instron 5569 Model, Norwood, MA, USA) equipped with a heating chamber. The following steps were performed: (1) The specimen with an initial length of L_0_ was heated to a constant programming temperature (T_p_) and maintained at that temperature for 5 min. It was then stretched to a specified% strain at a constant strain rate of 10 mm/min. (L_1_). This constraint was maintained while the specimen was quenched with an ice pack. (3) After removing the specimen from the test equipment, it was kept at room temperature for 24 h (L_2_). (4) The stretched specimens were immersed in a water bath at a specific recovery temperature (T_r_) to observe the recoverability of the specimen (L_3_). The test specimen collected at each stage of the shape memory test is illustrated in Figure 1. Tests were done on at least five specimens from each experimental condition. Shape recovery ratio (R_r_) and shape fixity ratio (R_f_) values of the specimens were determined as follows [39]:

(3) $$R_{r}(\%)=(\frac{L_{2}{-L}_{3}}{L_{2}{-L}_{0}})\times 100$$

(4) $$R_{f}(\%)=(\frac{L_{2}}{L_{1}})\times 100$$

The effects of compatibilizer concentration, and recovery temperature (T_r_) on the shape memory properties of PEG/PLA blends were investigated. To determine the effect of compatibilizer concentration, the specimens were stored at T_p_ of 45 °C and recovered at T_r_ 60 °C. To examine the effect of recovery temperature, specimens were kept at T_p_ of 45 °C before being immersed in a water bath at 40, 50 and 60 °C to observe their recoverability.

#### 2.4.5. Stress Relaxation Test

Stress relaxation test was conducted with the assistance of a Dynamic mechanical analyzer (DMA) (NETZSCH GABO EPLEXOR^®^ Serie ultra-high, Ahlden, Germany). The dimensions of a test specimen were 10 mm × 4 mm × 1.0 mm. Each specimen was kept at 45 °C for 5 min. It was then stretched to 100% strain at a rate of 10 mm/min. The tension was maintained throughout the experiment, and the stress reduction was plotted against time.

#### 2.4.6. Microstructure Evaluation

In situ Small-angle X-ray scattering and wide-angle X-ray scattering (SAXS/WAXS) measurements using BL1.3W beamline of Synchrotron Light, was applied to study microstructure evolution of specimens during shape memory test including the initial state, after heating, after stretching, and after recovery. X-ray energy of 9 keV (λ = 1.38 Å) was applied with the exposure duration of 60 s. q-range for SAXS measurement was 0.04–0.7 nm^−1^. The specimen-to-detector distances for WAXS and SAXS measurements were 0.24 and 5.18 m, respectively. WAXS and SAXS scattering data were processed by using the SAXSIT4.41 software (Synchrotron Light Research Institute, Nakhon Ratchasima, Thailand).

## 3. Results and Discussion

### 3.1. Characterizations of PLA-g-MA

**Figure 2:**
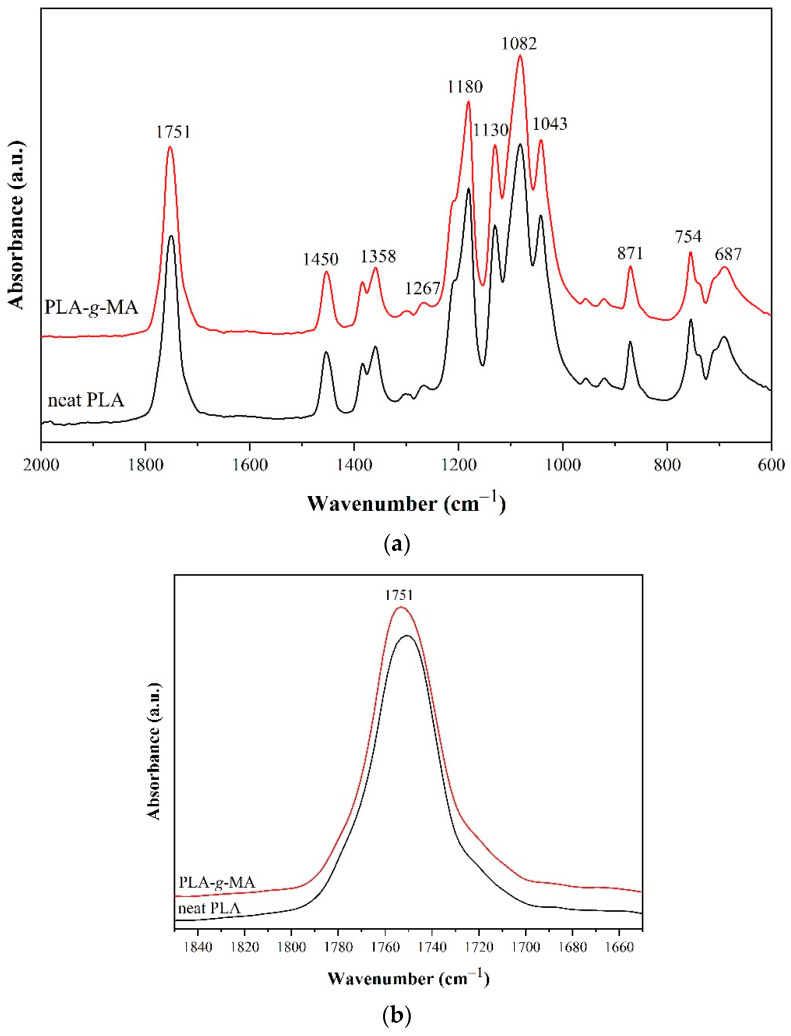

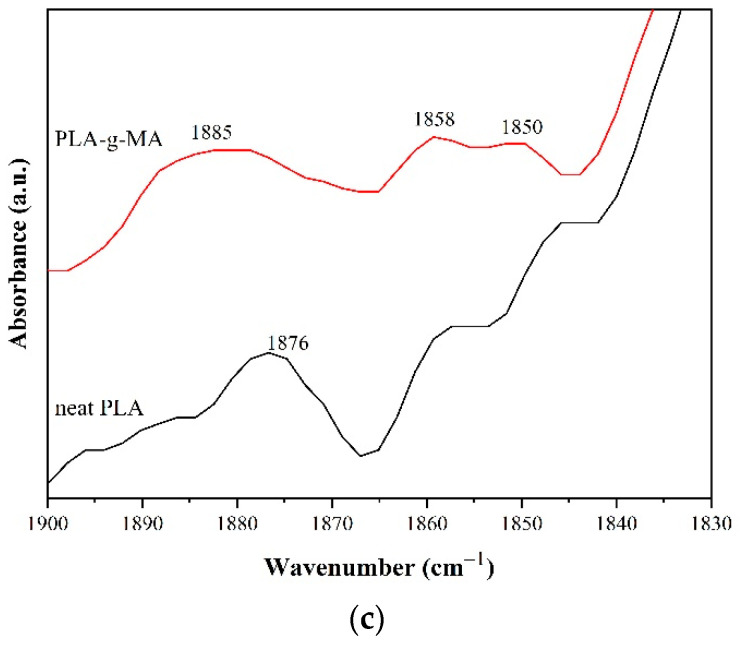
FTIR spectra of neat PLA and PLA-*g*-MA. (**a**) wavenumber range 2000–600 cm^−1^ (**b**) wavenumber range 1840–1660 cm^−1^ (**c**) wavenumber range 1900–1830 cm^−1^.

PLA-*g*-MA was synthesized through reactive blending to be utilized as a compatibilizer for PLA and PEG. Its grafting MA content was determined using titration technique, and the value was 0.45 wt%. The PLA-*g*-MA grafting reaction was also confirmed by FTIR, as shown in Figure 2. Due to the small amount of MA that has reacted with PLA chains, Figure 2a reveals minimal differences between the FTIR spectra of PLA and PLA-*g*-MA in the wavenumber range 2000–600 cm^−1^. The symmetrical carbonyl (C=O) stretching peak of PLA-*g*-MA around 1750 cm^−1^, in Figure 2b, shows a slight shift, peak broadening, and enhanced intensity in comparison to the C=O stretching peak of the neat PLA. This was due to the superposition of the C=O peaks of MA and PLA. Additionally, in Figure 2c, PLA-*g*-MA spectrum exhibits additional, broadening absorption bands in the range of 1885–1850 cm^−1^ as compared with those of the neat PLA. These corresponded to the asymmetric C=O stretching of the anhydride group of MA [35,36]. The results revealed the presence of reactive carbonyl groups of anhydrides in PLA-*g*-MA, which could indicate the occurrence of a PLA-*g*-MA grafting reaction.

### 3.2. Tensile Properties of PLA and PEG/PLA Blends with Various PLA-g-MA Contents

**Figure 3:**
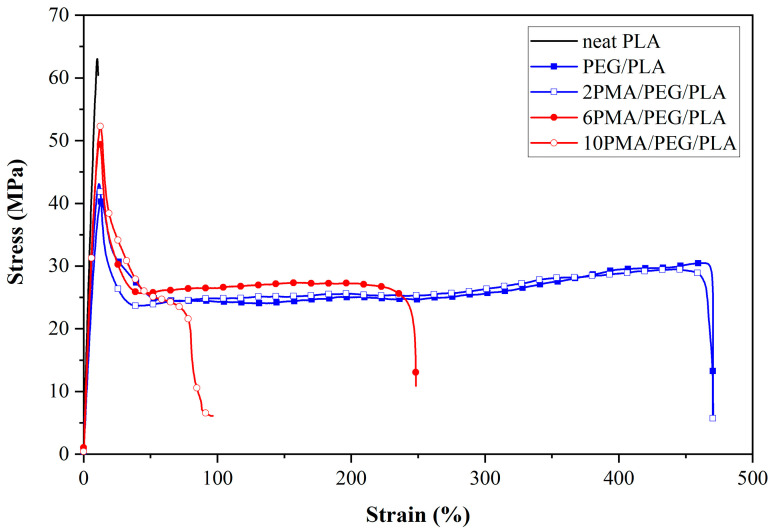
Stress-strain curves of PLA and PEG/PLA blends with various PLA-*g*-MA contents.

The stress-strain curves and summary of tensile properties of neat PLA and PEG/PLA blends with/without compatibilizer are shown in the Figure 3 and Table 1, respectively. As seen in Figure 3, neat PLA showed a brittle failure behavior. It possessed the highest Young’s modulus of 0.7 GPa and yield strength of 61.38 MPa, but very low elongation at break of 12.97%. The brittleness of PLA makes it unsuitable to be used for any shape memory application. By combining PLA with PEG, the ductility of the PLA was strengthened. The stress-strain curves of the uncompatibilized PEG/PLA blend exhibited a greater degree of plastic deformation than that of neat PLA. The breaking strain increased to up to 477%. Its Young’s modulus and yield strength expectedly decreased to 0.49 GPa and 40.22 MPa, respectively. This was the result of the plasticizing effect of PEG that allowed higher PLA chain mobility [28].

The addition of PLA-*g*-MA increased Young’s modulus and tensile strength of the compatibilized PEG/PLA blends, and the values increased with increasing PLA-*g*-MA content. The change in elongation at break of the 2PMA/PEG/PLA was statistically insignificant as compared to that of the uncompatibilized PEG/PLA. The results suggested that the existence of 2 wt% PLA-*g*-MA increased the compatibility of the PLA and PEG phases. The enhanced compatibility steamed from the grafting reaction between the anhydride groups of MA and hydroxyl groups of PLA similar to that reported by Hassouna et al. [36]. This interaction enhanced the interfacial adhesion by improving stress transfer around the interface, resulting in an increase in tensile strength while elongation at break remain unchanged. As PLA-*g*-MA concentrations increased up to 10 wt%, the elongation at break of the 6PMA/PEG/PLA and 10PMA/PEG/PLA significantly dropped. The plausible explanation was that an excess of PLA-*g*-MA was oversaturated at the interface. PLA-*g*-MA coalescence may form, consequently causing the decline of interfacial tension between the polymer components in the blend [40,41]. Similar findings were reported by Tang et al. [40] in the system of ethylene terpolymer as a compatibilizer of poly(ethylene terephthalate) (PET) and high-density polyethylene (HDPE) blends, and Liu et al. [41] in the system of maleated thermoplastic elastomer as a compatibilizer of polypropylene (PP)/polyamide-6 (PA6) blends.

### 3.3. Thermal Behaviors of PLA and PEG/PLA Blends with Various PLA-g-MA Contents

**Figure 4:**
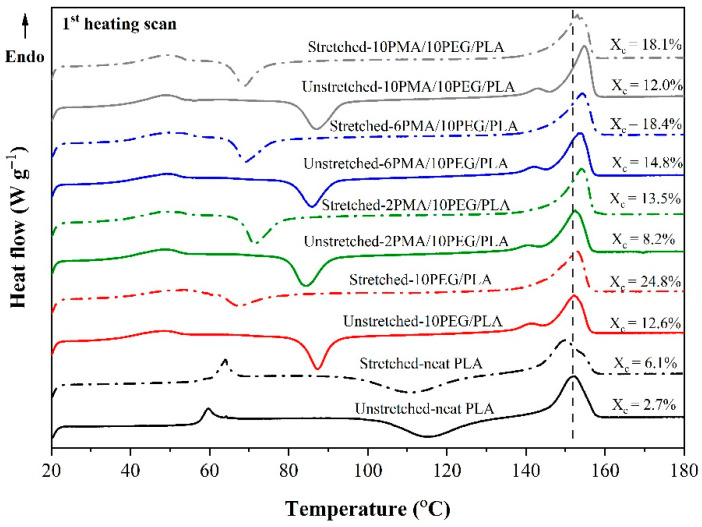
DSC thermograms of PLA and PEG/PLA blends in their unstretched and stretched states. Vertical line is used as a visual guideline for the shift of melting peak of the samples.

In this study, DSC technique was used to investigate the changes in thermal properties of the PLA and PEG/PLA blends at different stages of the shape memory test. This included testing the “unstretched sample” from the original shape specimen (L_0_) and the “stretched sample” from the temporary shape specimen (L_2_). Figure 4 depicts DSC thermograms of PLA and PEG/PLA blends in their unstretched and stretched states, and Table 2 is a summary of their thermal properties.

DSC thermogram of the neat, unstretched PLA showed T_g_ and cold crystallization temperature (T_cc_) at 58.3 °C and 115.5 °C, respectively. Because of the presence of 10 wt% PEG, the T_g_ and T_cc_ of the unstretched, uncompatibilized PEG/PLA were reduced to 40.3 °C, and 87.2 °C, respectively. This finding was the evidence of the plasticizing effect of PEG which enhanced polymer chain mobility and thus promoted PLA crystallization [28]. The addition of plasticizer also affected the crystal formation as the change of PLA melting peak was observed. The unstretched, uncompatibilized PEG/PLA exhibited a small endothermic peak at a temperature slightly below the main PLA melting peak, which was not present in the unstretched PLA. The small endothermic peak was attributed to the melting of PLA α’-crystals, while the main melting peak at 152.2 °C was attributed to the melting of PLA α-form crystals [42]. Moreover, the crystallinity (X_c_) of the PEG/PLA increased to 12.6% which was significantly higher than that of the PLA, which was just 2.7%.

When PLA-*g*-MA was added to the blends, the T_g_ values of the unstretched, compatibilized blends showed a slight upward trend as PLA-*g*-MA content increased. At 2 wt% PLA-*g*-MA loading, the T_cc_ value of the unstretched 2PMA/PEG/PLA decreased to 84.4 °C (from 87.2 °C of that of the uncompatibilized PEG/PLA) with no shift of the main T_m_ peak (152.4 °C). In addition, the X_c_ of the unstretched 2PMA/PEG/PLA was decreased to 8.2%, as compared to that of the uncompatibilized PEG/PLA (X_c_ = 12.6%). As the amount of PLA-*g*-MA was increased above 2 wt%, the T_cc_ and T_m_ of the compatibilized blends shifted to higher temperatures and the X_c_ of the 6PMA/PEG/PLA and 10PMA/PEG/PLA increased to 14.8% and 12%, respectively. This indicated that the addition of PLA-*g*-MA affected the lamellar structures of the compatibilized blends [37]. The addition of 2 wt% PLA-*g*-MA may improve the interfacial adhesion of the 2PMA/PEG/PLA, as a result of increased interfacial chain entanglement. This probably reduced the chain mobility of the PLA hence restricting crystallization [40]. However, as the PLA-*g*-MA content exceeded 2 wt%, the X_c_ of the compatibilized blends rose once again. This could be attributed to the PLA-*g*-MA coalescence serving as a nucleating agent in the compatibilized blends [41]. The DSC results were consistent with the tensile properties of the compatibilized blends that the elongation at break of the 6PMA/PEG/PLA and 10PMA/PEG/PLA significantly dropped as compared to that of the 2PMA/PEG/PLA.

In a separate experiment, DSC technique was also used to study effect of stretching on thermal properties and crystallinity of neat PLA and PEG/PLA blends. The test specimens were extended to 100% strain at 45 °C during the shape programming process then rapidly cooled down using ice packs. In the case of the neat PLA as shown in Figure 4, stretching caused an increase in T_g_ to 62.2 °C and a decrease in T_cc_ to 110.7 °C. In addition, the shape of the melting peak of the stretched PLA altered, and its T_m_ decreased to 149.9 °C while its X_c_ increased to 6.1%. This indicated that rapid PLA recrystallization and crystal disaggregation occurred when the specimen was extended at 45 °C, resulting a higher number of crystals [43].

The T_g_ of all stretched PEG/PLA blends, with and without PLA-*g*-MA, increased marginally. In comparison to their unstretched states, the stretching caused significant decreases in their T_cc_ and cold crystallization enthalpy (ΔH_cc_), whereas their crystallinity increased significantly. Moreover, all the stretched PEG/PLA blends showed a single endothermic peak, as opposed to the bimodal T_m_ peaks found in their unstretched states. Upon heating and stretching, crystallization occurred at a greater degree as the condition was more favorable for crystal formation. Furthermore, the recrystallization fully turned α’-form to α-form crystals after stretching [42], as evidently shown by an increase in the crystallinity and the altered shape of the melting peaks of the stretched PEG/PLA blends.

### 3.4. Morphologies of PLA and PEG/PLA Blends with Various PLA-g-MA Contents

**Figure 5:**
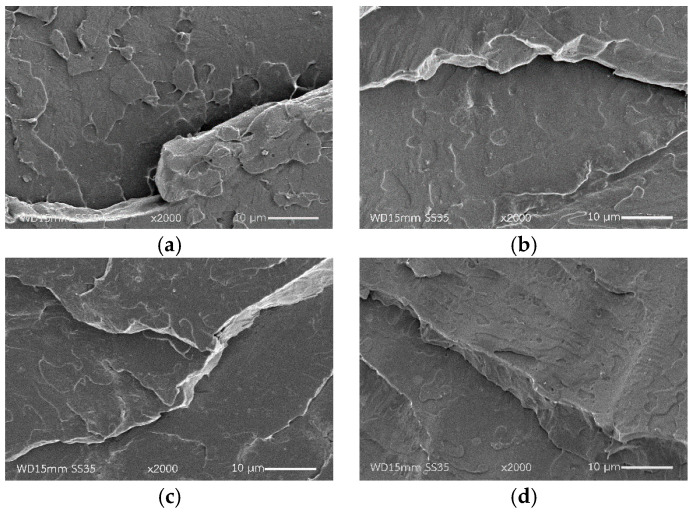

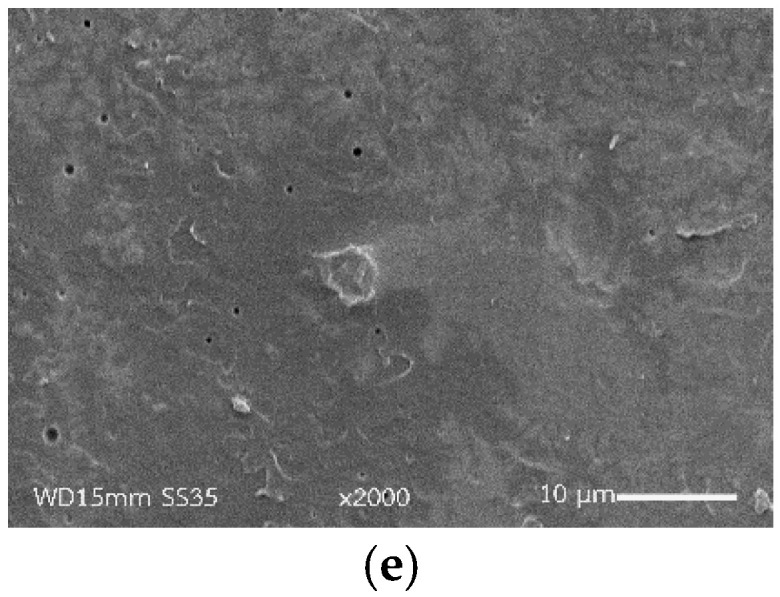
SEM micrographs of cryogenically fracture surfaces of (**a**) neat PLA, (**b**) PEG/PLA, (**c**) 2PMA/PEG/PLA, (**d**) 6PMA/PEG/PLA and (**e**) 10PMA/PEG/PLA.

**Figure 6:**
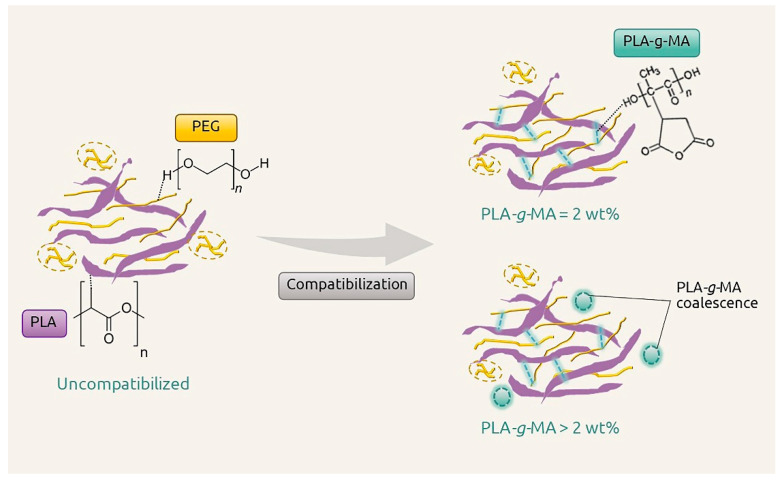
Schematic diagram for interaction between PLA, PEG, and PLA-*g*-MA.

Morphologies of PLA and PEG/PLA blends with various PLA-*g*-MA contents were examined using SEM. The results are shown in Figure 5. The SEM micrograph of the neat PLA in Figure 5a reveals a smooth surface of the cross-sectional area, whereas the micrograph of the uncompatibilized PEG/PLA in Figure 5b reveals a rough surface. The PEG minor phase dispersed in the continuous matrix of PLA in a non-spherical form without distinctive boundary or voids. This indicated the partial miscibility of the two polymers in the system. Several studies have found that PLA blended with PEG with a molecular weight of 1000–10,000 g/mol was found to be partially miscible at the critical PEG content of 10 wt% and became immiscible at the PEG content above 10 wt% [28,30,31]. The addition of 2 wt% PLA-*g*-MA caused no effect on the morphology of the 2PMA/PEG/PLA. However, as the compatibilizer content increased up to 6 wt% (Figure 5d), some agglomerations were observed in the morphology of the 6PMA/PEG/PLA. This could be the coalesces of the excess compatibilizer that led to the diminish in an interfacial interaction as mentioned in the tensile result. At the 10 wt% PLA-*g*-MA, in Figure 5e, the surface topography of the 10PMA/PEG/PLA showed a higher degree of agglomeration, with the microvoids easily visible. It can be inferred that when the compatibilizer content exceeded 2 wt%, there was a decline in the compatibility of the PEG/PLA blends. The schematic diagram of PEG/PLA blends with various PLA-*g*-MA contents is shown in Figure 6.

### 3.5. Shape Memory Behaviors of PLA and PEG/PLA Blends

The shape memory mechanism of the PEG/PLA blends can be explained by the work of the two major components in the system: i.e., switching phase or soft segment and fixing phase or hard segment. PEG and PLA chains in an amorphous region act as a switching phase. Physical chain entanglement and crystal of the blend act as netpoints or a fixity phase. In order to obtain highest shape fixity and shape recovery, the polymer chains in the amorphous phase must store the applied energy during the temporary shaping as much as possible. This could be possible if higher number of netpoints in the amorphous phase were available.

#### 3.5.1. Shape Memory Behaviors of the PLA and PEG/PLA Blends with Various PLA-*g*-MA Contents

**Figure 7:**
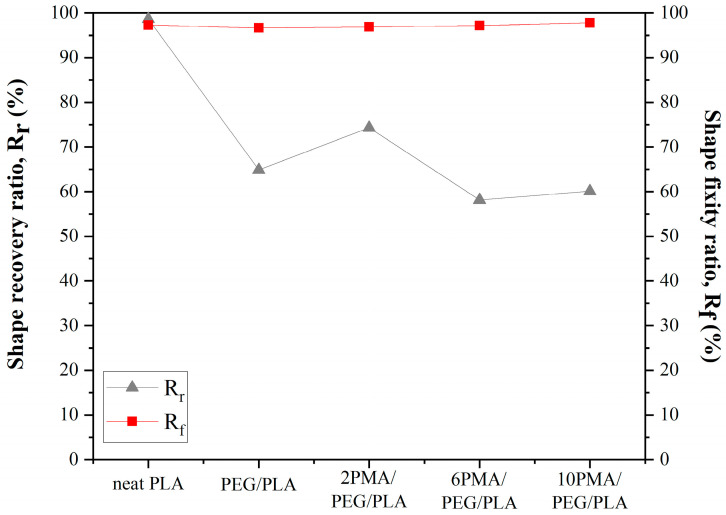
Shape recovery and fixity ratios of PLA and PEG/PLA blends.

Shape recovery (R_r_) and fixity (R_f_) ratios of neat PLA and PEG/PLA blends with various PLA-*g*-MA contents are shown in Figure 7. In this experiment, the programming and recovery temperatures were 45 and 60 °C, respectively, since these temperatures were slightly higher than T_g_ of the PEG/PLA blends.

R_r_ and R_f_ of the neat PLA were nearly perfect, exceeding 98%. All PEG/PLA blends with and without the PLA-*g*-MA compatibilizer demonstrated R_f_ values greater than 96%, indicating that the presence of PEG and PLA-*g*-MA had no obvious influence on the shape fixity ratio. When R_r_ was considered, the compatibilized PEG/PLA blend containing 2 wt% PLA-*g*-MA, 2PMA/PEG/PLA, had the highest R_r_ value among all the PEG/PLA blends. Further increased the amount of the compatibilizer to 6 wt% and 10 wt% caused the decreases in R_r_ values of the 6PMA/PEG/PLA and 10PMA/PEG/PLA. According to the DSC results of the PEG/PLA blends at various stages of the shape memory test, the stretching of the PEG/PLA blends resulted in a considerable increase in the crystallinity of the blends as compared to their corresponding unstretched states. This may account for the high R_f_ values seen in all PEG/PLA blends. Nonetheless, the stretched 2PMA/PEG/PLA had the lowest T_g_ and degree of crystallinity among the compatibilized PEG/PLA blends. This could be due to the enhanced interfacial adhesion in the 2PMA/PEG/PLA, which constrained the PLA crystallization process. Consequently, the PLA chains in 2PMA/PEG/PLA may have the highest tendency to return to their initial state at the recovery temperatures of 60 °C among the PEG/PLA blends. This gave 2PMA/PEG/PLA the highest R_r_ value. Guo et al. [28] also found that adding excess PEG to uncompatibilized PEG/PLA blends may cause irreversible deformation of the blends resulting in a reduction in R_r_.

#### 3.5.2. Shape Memory Behaviors of the PLA and PEG/PLA Blends at Various Recovery Temperatures

**Figure 8:**
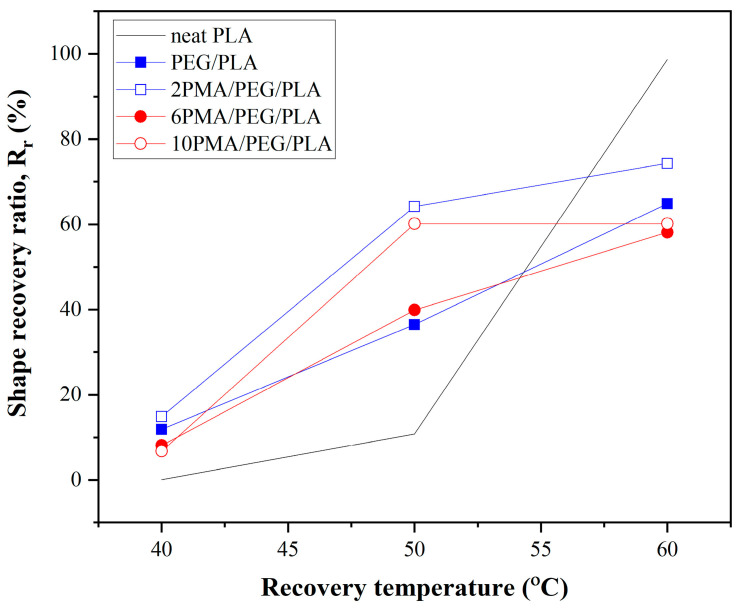
Shape recovery ratios of PLA and PEG/PLA blends at various recovery temperatures.

Additionally, the effect of recovery temperature (T_r_) on shape recoverability of PLA and PEG/PLA blends was examined. The stretched specimens were subjected to different temperatures of the water bath at 40, 50, and 60 °C to measure the recoverability. Their R_r_ values are illustrated in Figure 8.

For the neat PLA, the specimen could recover with a high R_r_ value at T_r_ of 60 °C. However, the neat PLA was difficult to recover at T_r_ below 60 °C. This was because its T_g_ was increased to 62 °C during shape programming process. This required more energy or heat to drive the recovery process. The PEG/PLA blends had the lower R_r_ as compared with the neat PLA. Nonetheless, all the PEG/PLA blends, with/without compatibilizer, could recover even at temperatures as low as 40 °C. Their R_r_ increased with the increasing T_r_. Among the PEG/PLA blends, 2PMA/PEG/PLA blend had the highest R_r_ values at all recovery temperatures. It was reported that a lowering of T_g_ can widen the recovery temperature of a shape memory polymer [28]. As the T_g_ of the PEG/PLA blends approached 40 °C, their onset recovery temperatures decreased. This could make them more appropriate for the use in biomedical applications.

### 3.6. Stress Relaxation of the PEG/PLA Blends with Various PLA-g-MA Contents

**Figure 9:**
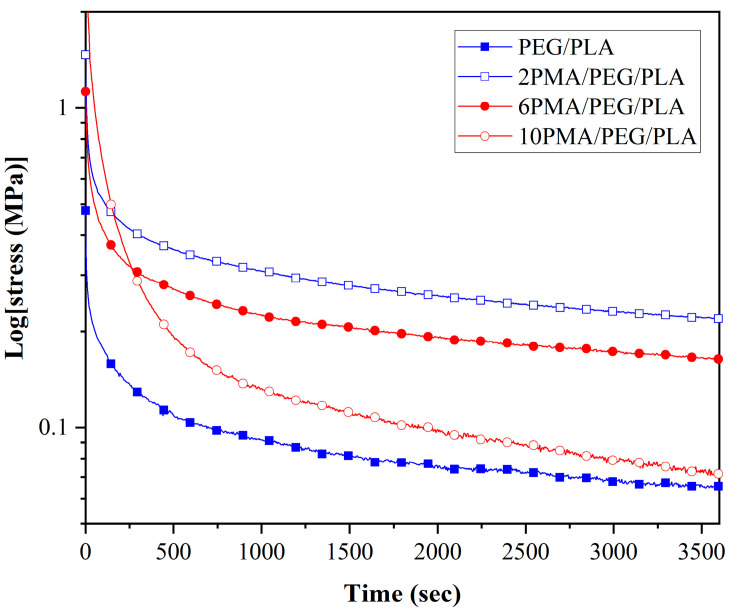
Stress relaxation of PLA and PEG/PLA blends at the shape programming temperature of 45 °C.

According to Tcharkhtchi et al. [44], the residual stress can be considered as the driving force for a SMP to regain its original shape. Shape recovery performance of the PEG/PLA blends can be explained by observing their stress relaxation behaviors at the shape programming temperature of 45 °C. The experiment was designed in a manner similar to the shape programming procedure; the specimen was first heated to 45 °C for 5 min before being stretched to 100% strain and monitored the stress reduction for 1 h. The stress relaxation curves of these specimens are illustrated in Figure 9.

As seen from the figure, 2PMA/PEG/PLA had the highest residual stress among the PEG/PLA blends. This could give the highest R_r_ value of the 2PMA/PEG/PLA. The residual stress of the compatibilized blends decreased as the added PLA-*g*-MA content exceeded 2 wt%. This was explained by the compatibilizer’s role in affecting the crystallinity of the blends. As previously stated, the presence of 2 wt% PLA-*g*-MA in the blend reduced the X_c_ and increased the chain entanglement of 2PMA/PEG/PLA as compared to that of the uncompatibilized PEG/PLA. Consequently, the 2PMA/PEG/PLA had a more difficult time releasing stress accumulated during temporary shape formation and a greater capacity to store driving stress energy for recovery to its original shape than the uncompatibilized PEG [45,46]. The addition of PLA-*g*-MA in excess of 2 wt% increased the X_c_, which lowered the recoverability of the blends. This result was consistent with DSC and shape memory test results.

### 3.7. Microstructures of PEG/PLA and 2PMA/PEG/PLA Blends during Shape Memory Test

**Figure 10:**
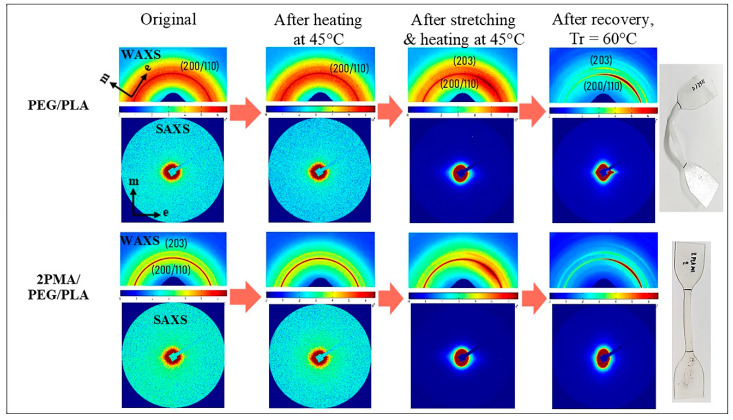
2D-WAXS and SAXS patterns of PEG/PLA and 2PMA/PEG/PLA specimens during shape memory test process.

In order to gain a better understanding of the function of PLA-*g*-MA in the PEG/PLA blends, in situ SAXS and WAXS measurements were used to determine the microstructure evolution of the uncompatibilized PEG/PLA and 2PMA/PEG/PLA during the programming and recovery processes. Scattering patterns of the blends are shown in Figure 10. As labeled in the figure, m and e represent the meridian and equator directions of the scattering profile, respectively. The stretching direction of the specimen was parallel to the meridian axis.

In the initial state, both uncompatibilized PEG/PLA and 2PMA/PEG/PLA exhibited unclear SAXS scattering patterns, indicating the random conformation of the polymer chains. The 2D-WAXS pattern of the uncompatibilized PEG/PLA demonstrated the uniform intensity of both the amorphous halo (orange color area) and the single ring of crystalline at (200/110) plane around the azimuth angle. The 2D-WAXS pattern of 2PMA/PEG/PLA demonstrated the isotropy of two distinct rings corresponding to (200/110) and (203) planes of α-form PLA crystallites [27]. These suggested that the polymer chains were isotopically oriented in the initial state. In addition, their WAXS and SAXS scattering patterns remained unchanged after heating these specimens at 45 °C.

After the shape programming process, the WAXS patterns of the stretched PEG/PLA and 2PMA/PEG/PLA specimens revealed a greater intensity of the crystallite ring along the equatorial axis, while their SAXS patterns revealed a small equator streak. These results suggested that polymer chains in both amorphous and crystalline regions were weakly anisotopically oriented perpendicular to the stretching direction during the shaping process. Since the specimens were deformed at low strain (100% strain), it might be insufficient to induce stacking lamellar perpendicular to the stretching direction [47]. The WAXS and SAXS patterns confirmed the DSC results that the polymer chains were oriented along the stretching direction, thereby increasing the crystallinity of the specimens.

**Figure 11:**
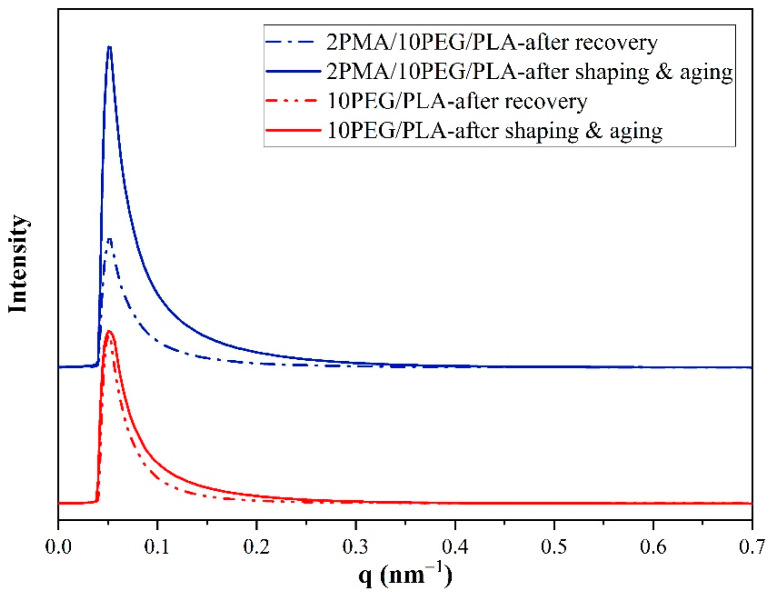
1D-SAXS plots along the equatorial axis of PEG/PLA and 2PMA/PEG/PLA specimens after aging and recovery at 60 °C.

After shape recovery at 60 °C, the equator streak in the SAXS pattern of the PEG/PLA had an asymmetrical appearance, which could be attributed to an unusual specimen recovery direction. In addition, the 1D-SAXS curves in Figure 11 demonstrate that the intensity of the equator streak in the 2PMA/PEG/PLA decreased significantly after recovery, while it remained unchanged in the PEG/PLA. The significant change in the intensity along the equator axis of the 2PMA/PEG/PLA may occur because this compatibilized specimen had less crystallinity or, in other words, the compatibilized blend contain relatively more polymer chains in the amorphous region than the uncompatibilized ones. This result explained why 2PMA/PEG/PLA exhibited greater residual stress than the PEG/PLA. When the 2PMA/PEG/PLA specimen was heated to 60 °C, the polymer chains in the amorphous region relaxed, hence restoring the chain to its original state. Consequently, the shape recovery ratio of the 2PMA/PEG/PLA was higher than that of the uncompatibilized PEG/PLA blend.

## 4. Conclusions

In this work, we investigated the role of PLA-*g*-MA in the PEG/PLA (10/90) blends in improving shape memory properties. The PEG (4000 g/mol) was added to PLA to reduce brittleness and lower recovery temperature to be in a useful range, close to physiological temperature. However, shape fixity and recovery ratio of the PEG/PLA blend were diminished. Poor compatibility was found to cause the decreases of the shape memory properties. The use of the PLA-*g*-MA as a compatibilizer in PEG/PLA was chosen to be a remedy for the issue. The presence of PLA-*g*-MA in the PEG/PLA blend caused the changes in mechanical and thermal properties, and shape memory behavior. The optimal content of the compatibilizer for PEG/PLA blend was found to be at 2 wt% which gave the highest tensile properties and lowest T_g_ and X_c_. The 2PMA/PEG/PLA possessed the maximum shape fixity and recoverability performance among the PEG/PLA blends at a programming temperature of 45 °C and a recovery temperature of 60 °C. The shape fixity performance of the compatibilized PEG/PLA blends was comparable to that of the neat PLA at the programming temperature of 45 °C. Although, a higher T_r_ was found to be beneficial for increasing recovery of the PEG/PLA blends, the compatibilized PEG/PLA blends can be recovered to their original shape at the temperature lower than the neat PLA. In situ SAXS and WAXS measurements revealed that higher number of oriented polymer chains in the amorphous region were relaxed in the 2PMA/PEG/PLA. Consequently, 2PMA/PEG/PLA possessed a higher shape recovery ratio than that of the uncompatibilized PEG/PLA. The finding was well agreed with DSC and stress relaxation results. In conclusion, PLA-*g*-MA as a compatibilizer plays a complex role in the shape memory behaviors of the PEG/PLA blends. PLA-*g*-MA provide anchor points for the two polymers in the partially compatible system of PEG/PLA blend. As optimum amount, PLA-*g*-MA could improve interfacial adhesion between phases, promote chain entanglement, and accumulate stress during the recovery process. Systematic studies are required in order to fully understand and utilize the polymer for its best performance.

## Figures and Tables

**Table 1:** Summarized tensile properties of PLA and PEG/PLA blends.

Sample	Tensile Strength [MPa]	Young’s Modulus [GPa]	Elongation at Break [%]
neat PLA	61.38 ± 1.24 ^#^	0.70 ± 0.02 ^#^	12.97 ± 0.92 ^#^
PEG/PLA	40.22 ± 0.60 *	0.49 ± 0.01 *	477.01 ± 62.03 *
2PMA/PEG/PLA	45.04 ± 1.32 *^,#^	0.53 ± 0.01 *^,#^	475.51 ± 88.87 *
6PMA/PEG/PLA	48.07 ± 0.90 *^,#^	0.55 ± 0.01 *^,#^	205.23 ± 73.74 *^,#^
10PMA/PEG/PLA	50.15 ± 1.53 *^,#^	0.56 ± 0.01 *^,#^	74.74 ± 11.78 ^#^

* *p* < 0.05, compared with the neat PLA. ^#^ *p* < 0.05, compared with the PEG/PLA.

**Table 2:** Summarized DSC results of PLA and PEG/PLA blends in their unstretched and stretched states.

Sample	T_g_ [°C]	T_cc_ [°C]	−ΔH_cc_ [J/g]	T_m_ [°C]	∆H_m_ [J/g]	Normalized X_c_ [%]
Unstretched-PLA	58.3	115.5	24.9	152.3	27.4	2.7
Unstretched-PEG/PLA	40.3	87.2	15.3	152.2	25.9	12.6
Unstretched-2PMA/PEG/PLA	41.6	84.4	18.4	152.4	25.2	8.2
Unstretched-6PMA/PEG/PLA	41.8	85.9	16.3	153.7	28.1	14.8
Unstretched-10PMA/PEG/PLA	42.3	87.1	18.0	154.9	29.3	12.0
Stretched-PLA	62.2	110.7	21.3	149.9	26.9	6.1
Stretched-PEG/PLA	43.8	67.5	8.6	152.8	29.5	24.8
Stretched-2PMA/PEG/PLA	41.8	71.3	16.7	154.1	27.9	13.5
Stretched-6PMA/PEG/PLA	42.6	69.0	13.0	154.2	27.7	18.4
Stretched-10PMA/PEG/PLA	42.8	68.8	12.4	153.2	29.4	18.1

## Data Availability

The data used to support the findings of this study are available from the corresponding author upon request.
